# The perception of intonational and emotional speech prosody produced with and without a face mask: an exploratory individual differences study

**DOI:** 10.1186/s41235-022-00439-w

**Published:** 2022-10-04

**Authors:** Chloe Sinagra, Seth Wiener

**Affiliations:** Language Acquisition, Processing, and Pedagogy Lab, Department of Modern Languages, Carnegie Mellon University, Pittsburgh, PA USA

**Keywords:** Face masks, Speech perception, Prosody, Intonation, Emotion, Individual differences, Autism, Memory

## Abstract

Face masks affect the transmission of speech and obscure facial cues. Here, we examine how this reduction in acoustic and facial information affects a listener’s understanding of speech prosody. English sentence pairs that differed in their intonational (statement/question) and emotional (happy/sad) prosody were created. These pairs were recorded by a masked and unmasked speaker and manipulated to contain audio or not. This resulted in a continuum from typical unmasked speech with audio (easiest) to masked speech without audio (hardest). English listeners (*N* = 129) were tested on their discrimination of these statement/question and happy/sad pairs. We also collected six individual difference measures previously reported to affect various linguistic processes: Autism Spectrum Quotient, musical background, phonological short-term memory (digit span, 2-back), and congruence task (flanker, Simon) behavior. The results indicated that masked statement/question and happy/sad prosodies were harder to discriminate than unmasked prosodies. Masks can therefore make it more difficult to understand a speaker’s intended intonation or emotion. Importantly, listeners differed considerably in their ability to understand prosody. When wearing a mask, speakers should try to speak clearer and louder, if possible, and make intentions and emotions explicit to the listener.

## Significance statement

For surgeons and painters, communication in face masks is common. For others, COVID-19 marked the beginning of talking (speech production) and listening (speech perception) while wearing a mask. Masks can affect the transmission of the speech signal and obscure facial cues. This change in listening conditions has affected people differently. What are some of the factors that cause this individual variability in listeners? This study explored that question in terms of speech prosody. The utterance “it’s raining” can be a statement (flat intonation) or a question (rising intonation). Prosody is often accompanied with facial cues, such as head tilts and eyebrow raises. Masks can muffle speech cues and hide facial cues, which can make prosody difficult to understand. Our study found that masks make it harder to understand a speaker’s statement/question intonational prosody and happy/sad emotional prosody. Among the individual differences we tested, we found that Autism Spectrum Quotient predicted some performance on the prosody discrimination task. The findings have potential educational and clinical implications. When speaking with a mask, speakers should increase pitch and volume, if possible. Because facial cues may be obscured, speakers should also be more explicit about their intended emotions/questions (e.g., “I’m happy it’s raining.” “I have a question: is it raining?”).

## Introduction

To fight the spread of the COVID-19 virus, facial mask mandates were put in place by governments throughout the world. For many people, this was the first time both the speaker and listener wore masks during communication. Masks have acoustic and visual consequences. Acoustically, the materials made to reduce the transmission of pathogens also reduce sound transmission (Magee et al., 2020). As a result, masks can reduce a speaker’s fundamental frequency (*F*0: what listeners perceive as pitch) and amplitude (what listeners perceive as volume or loudness). For many listeners, this reduction in acoustic information makes understanding speech more difficult (e.g., Brown et al., 2021; Fiorella et al., 2021; Mheidly et al., 2020). Visually, a mask obscures the mouth and hides facial cues. Visual information like mouth movements can help a listener better understand acoustic information (e.g., Best, 1995; Fowler, 1986; Saunders et al., 2021). For example, the relatively similar sounding English speech sounds /s/ and /ʃ/ differ in their lip-rounding, which listeners can use to better understand whether the speaker needs to *sip* the bottle or *ship* the bottle. For those listeners with hearing problems, communicating in noisy environments, and listening to non-native speech, visual cues can be very helpful (Fiorella et al., 2021; House et al., 2001; Sueyoshi & Hardison, 2005; Winn et al., 2013).

In the present study, we extend recent research into masks and speech perception by examining the perception of speech prosody and masks. Prosody is a broad term that includes pitch, stress, rhythm, and intonation (e.g., Cutler, 2012; Cutler et al., 1997). It is often described as not *what* a speaker says, but *how* it is said. For example, a student telling a friend, “Class is cancelled” could convey happiness because it is a boring class or sadness because it is the student’s favorite class. Acoustic cues like *F*0 and amplitude (among others) change given the prosody of the speech. Here, we examine intonational statement/question prosodies and emotional happy/sad prosodies produced with and without masks. Statements are usually characterized by their relatively falling volume and pitch, whereas questions are usually characterized by their relatively rising volume and pitch (Gussenhoven & Chen, 2000; Pell, 2001; Srinivasan & Massaro, 2003). Happy speech is typically characterized by its relatively high volume and high pitch; in contrast, sad speech is typically characterized by its relatively low volume and low pitch (Bänziger & Scherer, 2005; Scherer, 2003; Sobin & Alpert, 1999).

Smiling, frowning, and raising and lowering the head regularly accompany speech prosody (Graf et al., 2002; Granström & House, 2005; Granström et al., 1999). Listeners can use these facial cues to better understand the speaker’s intent and emotions (Lansing & McConkie, 1999; Munhall et al., 2004). Masks can obscure some of these cues, which makes detecting a speaker’s emotions more difficult (Carbon, 2020). Yet, adults differ considerably in their ability to interpret these visual cues (e.g., Gandour et al., 2003; Lambrecht et al., 2012; Rymarczyk & Grabowska, 2007). This individual variation in prosody perception (e.g., Cole, 2015; Ward, 2019) is the focus of our study. Here, we examine how listeners differ in their perception of intonational (statement/question) and emotional (happy/sad) prosody in which the target sentences contain identical words, but contrast in their perceived loudness and pitch, as well as subtle facial cues.

Given that COVID-19 made laboratory-based data collection difficult, we collected data on behavioral differences using short, reliable tasks easily administered via the internet. This extends previous laboratory-based research on the individual differences in listeners’ perception of prosody (e.g., Ferreira & Karimi, 2015; Jun & Bishop, 2015) by testing a larger, more diverse sample size beyond the laboratory. We manipulated the presence of masks and audio to create a continuum from typical unmasked speech with audio (easiest), to masked speech without audio (hardest). We also examined four measures previously reported to affect language processes: Autism Spectrum Quotient, musical background, phonological short-term memory, and congruence task behavior.

Individuals diagnosed with autism spectrum disorder tend to struggle with the perception of prosody, recognition of emotions, and overcoming face masks (see McCann & Peppé, 2003 for a review). In particular, adult listeners diagnosed with autism spectrum disorder struggle to recognize the facial and acoustic cues associated with emotions like happy and sad (e.g., Clark et al., 2008; Peppé et al., 2011; Philip et al., 2010). We predict those listeners with greater “autistic” traits will be less accurate at discriminating prosody than those listeners with fewer “autistic” traits. This difference may be particularly noticeable in the happy/sad prosody given that facial cues typically accompany this speech, and in masked speech given that facial cues are further reduced.

Music and language are believed to be processed in similar parts of the brain given their shared use of voice and rhythm (Patel, 2010). Musicians typically outperform non-musicians in a wide range of prosody perception tasks involving rhythm, stress, tone, and emotion (e.g., Hausen et al., 2013; Lima & Castro, 2011; Thompson et al., 2004). This is typically attributed to musicians’ greater sensitivity to pitch (*F*0 cues) and volume (amplitude cues) as a result of their training. We predict those listeners with greater musical training will more accurately discriminate happy/sad and statement/question prosody than those listeners with less or no training. Musicians may also show an advantage over non-musicians in masked speech given their greater sensitivity to *F*0 and amplitude cues.

Phonological short-term memory affects a wide range of linguistic processes, including prosody recall, discrimination, and categorization (e.g., Baddeley et al., 1984; Jacquemot & Scott, 2006; Lambrecht et al., 2012; Stepanov et al., 2020). Because accurate perception of prosody requires not only accessing the meaning of the words (phonological sound-to-meaning mapping), but also recognizing how that meaning may change given variations in the acoustics heard at a later point in time (see Cutler, 2012 for a review), a listener with greater phonological short-term memory may be able to better store meaning and acoustics than a listener with weaker phonological short-term memory. This may be especially helpful for overcoming masked speech, which typically dampens prosodic cues like pitch and volume. We predict that individuals with greater phonological short-term memory will more accurately discriminate prosody than those with lesser phonological short-term memory.

Finally, listeners differ in their ability to focus on the task at hand when distracted. A large body of psychometric literature has examined how the ability to resist distractor interference and inhibit pre-potent responses contributes to human behavior (often discussed as “cognitive inhibition,” see Lu & Proctor, 1995; Rey-Mermet, Gade, & Oberauer, 2018). Many congruency tasks in which the participant must ignore and suppress irrelevant or incongruent information have been found to predict performance on a variety of linguistic tasks, especially tasks involving switching between different languages and linguistic units (e.g., Blumenfeld & Marian, 2014; Pliatsikas & Luk, 2016). We note, however, which specific congruence task is used and what linguistic construct (if any) it predicts, varies considerably across the literature (e.g., Hedge et al., 2018; Poarch & Van Hell, 2012). Here, we carry out exploratory research to examine whether behavior on two congruency tasks (one linguistic and one non-linguistic) can predict prosody discrimination performance. We predict that listeners who perform better on congruency tasks, that is, are better able to focus on the task despite incongruent information, will more accurately discriminate prosody than those who perform poorly on the congruency task and are unable to focus on the task given incongruent information. This difference may be particularly robust in masked speech, which can confuse the listener in terms of reading emotions (Carbon, 2020).

## Methods

All stimuli, data, and R code are available on the Open Science Framework.

https://osf.io/gs79t/.

### Participants

The experiment was built and run using Gorilla (Anwyl-Irvine et al., 2020). A total of 165 participants were initially recruited from Prolific (www.prolific.co). All participants were 18 years of age or older (mean = 32.8) with normal hearing. All participants self-identified as monolingual English speakers who had learned English from birth. Participants were required to use only a desktop or laptop computer. Participants were asked to wear headphones and confirm that they would wear them during the entire experiment. The experiment took roughly 30 min to complete. Participants were paid for their time ($11/h). The experiment was approved by the Carnegie Mellon University Institutional Review Board. From the original 165 participants tested, 16 were removed for having hearing problems, 5 for failing attention checks, 5 for data failure, and 11 for below chance performance in either the happy/sad or statement/question prosody task. This left data from 128 participants, which we report below.

### Questionnaires

Participants completed two questionnaires: Autism Spectrum Quotient and Music Use. The Autism Spectrum Quotient (Baron-Cohen et al., 2001) is a brief questionnaire containing 50 questions across five areas: social skill, attention switching, attention to detail, communication, and imagination. Each question allowed four choices (definitely agree, slightly agree, slightly disagree, definitely disagree) and therefore allowed for an estimation of autism spectrum traits. Following Baron-Cohen et al., each autistic-like behavior was scored as 1 (irrespective of whether it was a “definitely” or “slightly” response) whereas each non-autistic-like behavior was scored as 0 (irrespective of whether it was a “definitely” or “slightly” response). This resulted in a total score for each participant ranging from 0 to 50 with higher scores reflecting greater autistic-like behavior; scores of 32 or greater represented what Baron-Cohen et al. call “a useful cutoff for distinguishing individuals who have clinically significant levels of autistic traits” (2001, 15). The internal consistency of the questions, as measured by Cronbach’s alpha, was 0.87, or “good” (Cronbach, 1951).

Music Use (MUSE: Chin & Rickard, 2012) is a brief questionnaire containing questions aimed at measuring levels of music listening, training, and instrument playing, in addition to music engagement and experience. It contains eight open ended and 24 Likert scale questions. For the purposes of our study, Music Use scores were calculated for each participant as a summary score across the 24 Likert scale questions. Each Likert scale question allowed for six choices (not applicable to me, strongly disagree, disagree, neither agree nor disagree, agree, strongly agree) corresponding to a 0 to 5 value. This resulted in a total score for each participant ranging from 0 to 120 with higher scores reflecting greater engagement and experience with music. The internal consistency of the items was “excellent” (Cronbach’s *α* = 0.93; Cronbach, 1951).

### Phonological short-term memory tasks

Participants completed two phonological short-term memory tasks: digit span (e.g., Jacquemot & Scott, 2006) and 2-back (e.g., Kane et al., 2007). These two tasks involved briefly presenting a stimulus and asking the participant to recall it later. Participants were told to remember the presented information as they would be asked about it later, but not to write anything down.

The digit span task presented 10 increasingly long sequences containing one digit (first sequence) to 10 digits (tenth sequence). Participants were shown each digit for 2000 ms followed by a 100 ms fixation with consecutive digits repeating in that pattern. After each sequence was presented with all its digits, participants were asked to type the numbers in the correct order. The largest sequence with all its digits correctly recalled was calculated for each participant (1–10), i.e., the “highest score” method, which typically yields higher reliability than a total score across all trials (Groth-Marnat & Baker, 2003). A larger number represented a greater phonological short-term memory. The internal consistency of the items was “acceptable” (Cronbach’s *α* = 0.70; Cronbach, 1951).

The 2-back task presented 32 English letters one at a time for 2000 ms each. After each letter was presented, participants were asked if that letter was presented two trials ago. There were nine targets and 23 incorrect foils. If the letter was presented two trials ago, participants were asked to press the “F” key; if the letter was not, participants were asked to press the “J” key. A 2000 ms response time limit was given for each letter and the next letter was displayed immediately after a button press. Because the first two trials were not scored, participants’ scores ranged from 0 to 30 and represented the correct total trials. A larger number represented a greater phonological short-term memory. The internal consistency of the items was “good” (Cronbach’s *α* = 0.89; Cronbach, 1951).

### Congruency tasks

Participants completed two congruency tasks: flanker (Eriksen & Eriksen, 1974) and Simon (see Lu & Proctor, 1995). These two tasks presented multiple stimulus–response congruency trials. On each trial, information was either congruent or incongruent and required a keyboard press from the participant.

The flanker task showed five cartoon fish in a row with the middle fish either facing the same direction as the others (congruent) or facing the opposite direction (incongruent). Participants were asked to press “F” if the middle fish was swimming to the left; “J” if it was swimming to the right. Four practice trials with feedback were presented, followed by 36 trials without feedback. Of the 36 trials, 18 were congruent (9 swimming left; 9 swimming right) and 18 were incongruent (9 swimming left while others swam right; 9 swimming right while others swam left). A 2000 ms response time limit was given for each trial. Each trial immediately advanced upon button press. The internal consistency of the items was “excellent” (Cronbach’s *α* = 0.94; Cronbach, 1951). Response time results were calculated by first removing incorrect trials (1%), and then calculating the Median Absolute Deviation using the *psych* package in R (Leys et al., 2013). Outliers were defined as the median plus or minus three times the Median Absolute Deviation. Roughly 8% of the data were removed as outliers. The remaining correct response times from congruent trials were subtracted from those of incongruent trials, resulting in a mean RT difference for each participant. A larger RT difference reflected worse abilities to ignore the incongruent information whereas a smaller RT difference reflected better abilities to ignore incongruent information and focus on the task.

The Simon task showed the words “left” and “right” on the two sides of the computer screen. Participants were asked to press the “F” key if the word “left” appeared on the screen and the “J” key if the word “right” appeared, irrespective of location. Four practice trials with feedback were presented, followed by 36 trials without feedback (18 congruent, i.e., “left” on the left-hand side and “right” on the right-hand side; 18 incongruent, i.e., “left” on the right-hand side and “right” on the left-hand side). The words “left” and “right” each appeared 18 times. A 2000 ms response time limit was given for each trial. The internal consistency of the items was “poor” (Cronbach’s *α* = 0.55; Cronbach, 1951). Response time results were calculated by first removing incorrect trials (9%), and then calculating the Median Absolute Deviation using the *psych* package in R (Leys et al., 2013). Outliers were defined as the median plus or minus three times the Median Absolute Deviation. Roughly 5% of the data were removed as outliers. As with the flanker task, remaining correct response times from congruent trials were subtracted from those of incongruent trials, resulting in a mean RT difference for each participant. A larger RT difference reflected worse abilities to ignore the incongruent information whereas a smaller RT difference reflected better abilities to ignore incongruent information and focus on the task.

### Prosody task

**Fig. 1:**
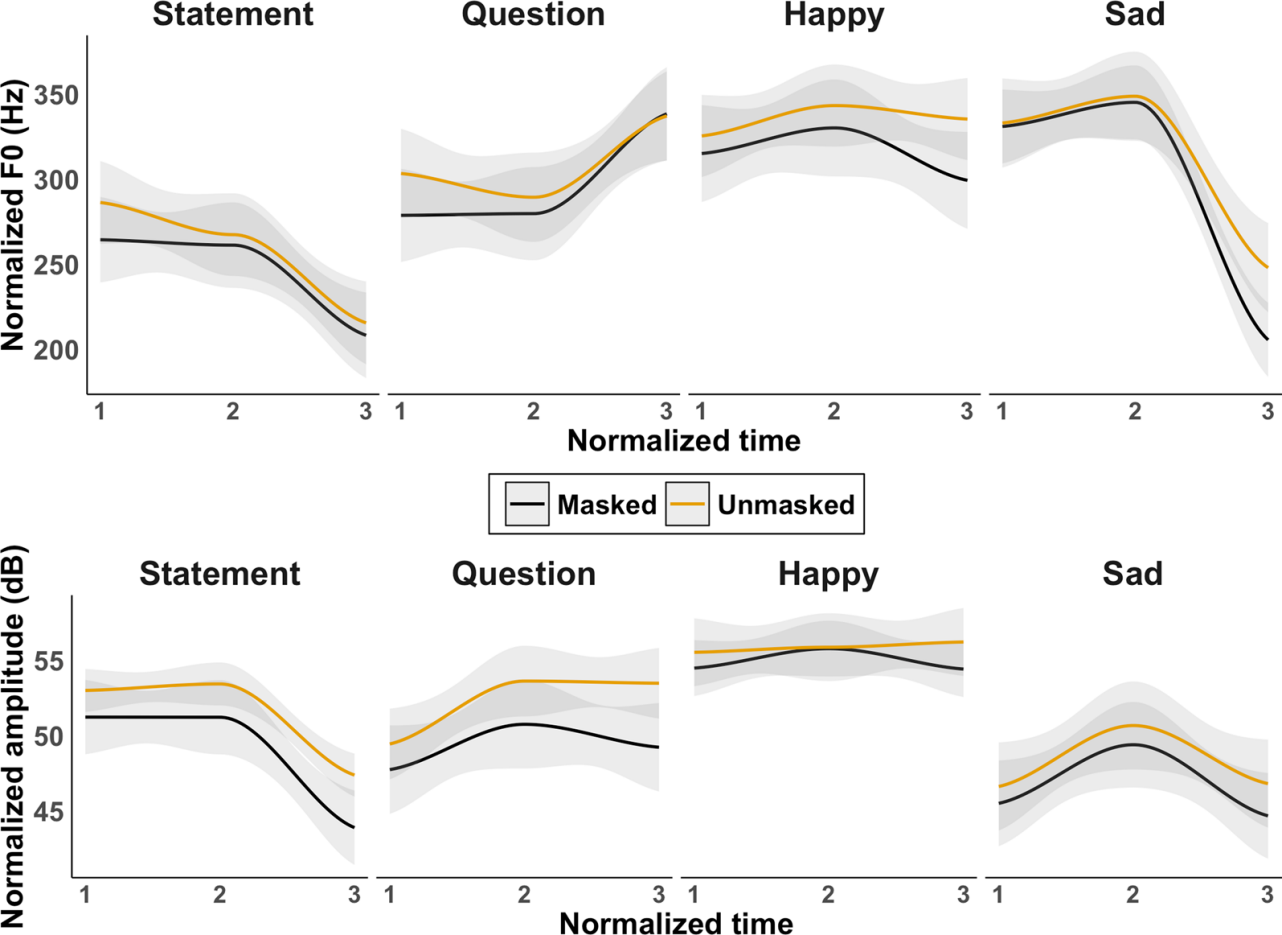
Normalized *F*0 (top) and amplitude (bottom) over three normalized time points. Solid lines represent a smoothed best fit with gray 95% confidence intervals

Thirty-two statement/question (e.g., “Class is cancelled”) and 32 happy/sad (e.g., “It’s time for class”) sentences were created. These sentences were designed to be concise, natural utterances someone might say, with easy-to-understand content. This resulted in 128 unique items (32 statement + 32 question + 32 happy + 32 sad). Half of the sentences (16 per prosody type) were recorded unmasked and half with a cloth face mask worn securely on the face (black, filterless). Recording was done in a quiet room with a blank background using an iPad Air (4th Generation), placed roughly 2 feet in front of the speaker. The speaker was a 20-year-old female university student who spoke American English. The videos were filmed in two sessions corresponding to the prosody: question/sentence and happy/sad. Each sentence was said twice in succession, with a fixed order of question–statement or happy–sad. The 128 videos were cut into individual files, each approximately 2–4 s long and saved as an mp4. The audio was recorded at 44.1 kHz using the internal microphone from the iPad. The audio was unaltered for the purposes of the study. Each item was labeled and analyzed in Praat (version 6.1.08; Boersma & Weenink, 2021) with all normalized measurements obtained using ProsodyPro (Xu, 2013). Figure 1 plots the normalized *F*0 and amplitude measurements over three normalized time points. The solid line represents a smoothed best fit with gray 95% confidence intervals. This figure shows that masked speech had a lower overall normalized mean *F*0 and amplitude than unmasked speech.

Statistically modeling prosody with or without a time variable can be done using different approaches (see Xu & Prom-on, 2015). We were interested in demonstrating a difference between statement and question prosodies, happy and sad prosodies, and speech produced with and without a mask. We therefore calculated an overall mean (across the three normalized time points) for each of the 128 items. For *F*0, a two-way ANOVA confirmed differences between masked and unmasked speech [*F*(1, 120) = 6.27, *p* = 0.01, *η*_p_^2^ = 0.05], and prosody [*F*(3, 120) = 29.19, *p* < 0.001, *η*_p_^2^ = 0.42], but no two-way interaction (*p* = 0.95). The same pattern was found for amplitude: masked [*F*(1, 120) = 9.76, *p* = 0.002; *η*_p_^2^ = 0.08], prosody [(*F*(3, 120) = 28.21, *p* < 0.001; *η*_p_^2^ = 0.41)], null two-way interaction (*p* = 0.68). Tukey-adjusted pairwise comparisons confirmed that the prosody between statement and question items and happy and sad items differed from one another in mean *F*0 and amplitude (*p*s < 0.05). Although masked speech lowered mean *F*0 and amplitude overall, after correcting for multiple comparisons no difference was found in any of the eight individual comparisons (*p*s > 0.05). In other words, the decrease in *F*0 and amplitude were aggregate effects across all masked items.

An additional set of 128 videos were created by removing the audio track with the program *ffmpeg* (Tomar, 2006). This resulted in a total of 256 items (128 with audio + 128 without audio). From these 256 items, two half lists of the 128 items were created. This kept the prosody task under 15 min, guarded against potential boredom, and meant that each participant heard a sentence in only one type of prosody, not both, and as either masked or unmasked, not both. For example, in half list 1 the statement, “You burned it.” was presented with audio and the question, “You burned it?” was presented without audio. In half list 2, the question was presented with audio and the statement without audio. These two half lists contained eight different prosody–mask–audio blocks (2 × 2 × 2) with 16 items in each block (8 × 16 = 128 items). The order of the eight blocks was counterbalanced using a Latin-square design.

**Fig. 2:**
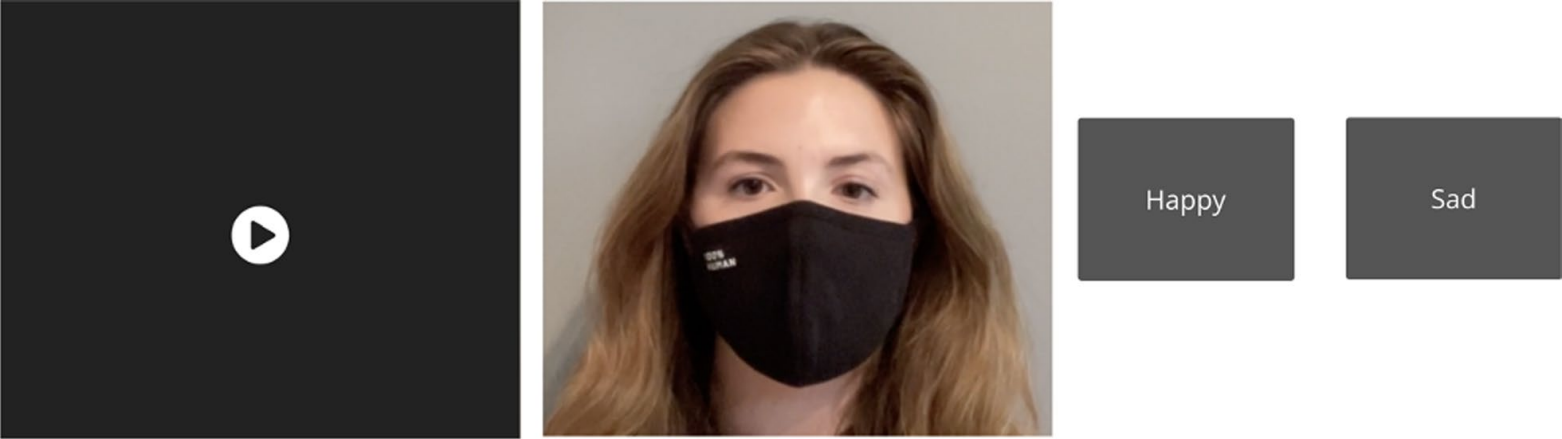
Prosody task sequence from left to right: mouse-click play button, video played only once, prosody options

Before beginning the 8 blocks, participants completed practice trials explaining the task and asking the listener to find a suitable volume. At the start of each block participants were told whether there would be audio or not, and to click as quickly and accurately as possible on the perceived statement/question or happy/sad intonation. In each block, a video was first presented, which could only be played once (Fig. 2, left). After mouse-clicking the play button, the video began (Fig. 2, center). Participants were then presented with the prosody choices (Fig. 2, right). Location of each prosody button was counterbalanced across all trials. Participants who did not perform above chance (0.5) in either the happy/sad or statement/question condition (*N* = 11) were removed from the data.

## Procedure

Participants completed the experiment in a fixed order: IRB information and consent, flanker task, Autism Spectrum Quotient questionnaire, digit-span task, Music Use questionnaire, Simon task, prosody task, 2-back task. The entire procedure took approximately 30 min to complete. An attention check occurred after three tasks and six tasks, respectively. In this attention check, participants were required to click on a button five times before progressing to the next task. Participants who failed to click on the button five times within a minute of presentation (at either check) were removed (*N* = 5).

### Data analysis

All analyses were carried out in R (version 4.1.0; R Core Team, 2020) with a 0.05 alpha level. The accuracy of the prosody task (coded as 1 correct; 0 incorrect) was modeled using generalized linear mixed-effects models with a logit link function using the *lme4* package (version 1.1.29). Two separate models were run corresponding to the prosody: statement/question and happy/sad. The fixed effects included the three experimental manipulations of mask, audio, and prosody, all of which were dummy coded with the reference levels as: “unmasked,” “audio,” and “question prosody” or “happy prosody.” This meant the inclusion of a mask in the “masked” condition should reduce the log-odds of correct identification (as reflected by a negative coefficient), and the removal of audio in the “no audio” condition should reduce the log-odds of correct identification (as reflected by a negative coefficient). Any difference between prosodies relative to the “question” or “happy” prosody will be reflected by a positive (i.e., accuracy increase) or negative (i.e., accuracy decrease) coefficient.

The scores from the six individual differences tasks were first standardized and then included as fixed effects in the models. Positive coefficients reflect an increase in log-odds of correct identification (given a one-unit increase for the variable) whereas negative coefficients reflect a decrease in log-odds of correct identification (given a one-unit increase for the variable). For example, a positive coefficient for Autism Spectrum Quotient will indicate that more autistic-like behavior resulted in an increase in log-odds of correct identification (i.e., accuracy increase) whereas a negative coefficient will indicate that more autistic-like behavior resulted in a decrease in log-odds of correct identification (i.e., accuracy decrease). For Music Use, digit span, and 2-back tasks, we expect a positive coefficient as better music abilities and better phonological short-term memory should lead to more accurate identification. For Autism Spectrum Quotient, we expect a negative coefficient as more autistic-like behavior should lead to less accurate identification. For flanker and Simon tasks, we also expect a negative coefficient as a larger difference between congruent and incongruent trials (i.e., greater incongruity costs) should lead to poorer identification accuracy.

For each analysis, the maximal model was first fit. The maximal model contained fixed effects of mask, audio, prosody, and all six individual differences measures. Both two-way and three-way interactions were included in the model. Because mask, audio, and prosody were not manipulated within-items, and because any individual difference effect should be constant by participant (see Barr et al., 2013 and Brown, 2021 for discussions), the model therefore contained by-subject random slopes for mask, audio, prosody and by-item random slopes for all six individual difference tasks. If this model did not converge or produced a singular fit, random slopes that contributed the least amount of variance were removed until the model converged without a singular fit. Model quality (conditional *R*^2^ and Bayesian Information Criterion) was assessed using the *performance* package (Lüdecke et al., 2021). The final statement/question model:

(accuracy ~ mask * autism quotient + autism quotient * prosody + audio * autism quotient + simon * mask + simon * prosody + audio * simon + 2-back * mask + 2-back * prosody + audio * 2-back + digit span * mask + digit span * prosody + audio * digit span + flanker * mask + flanker * prosody + audio * flanker + music use * mask + music use * prosody + audio * music use + mask * audio * prosody + (autism quotient | item) + (mask + prosody + audio | participant)).

The final happy/sad model:

(accuracy ~ mask * autism quotient + autism quotient * prosody + audio * autism quotient + simon * mask + simon * prosody + audio * simon + 2-back * mask + 2-back * prosody + audio * 2-back + digit span * mask + digit span * prosody + audio * digit span + flanker * mask + flanker * prosody + audio * flanker + music use * mask + music use * prosody + audio * music use + mask * audio * prosody + (2-back | item) + (mask + prosody + audio | participant)).

## Results

**Fig. 3:**
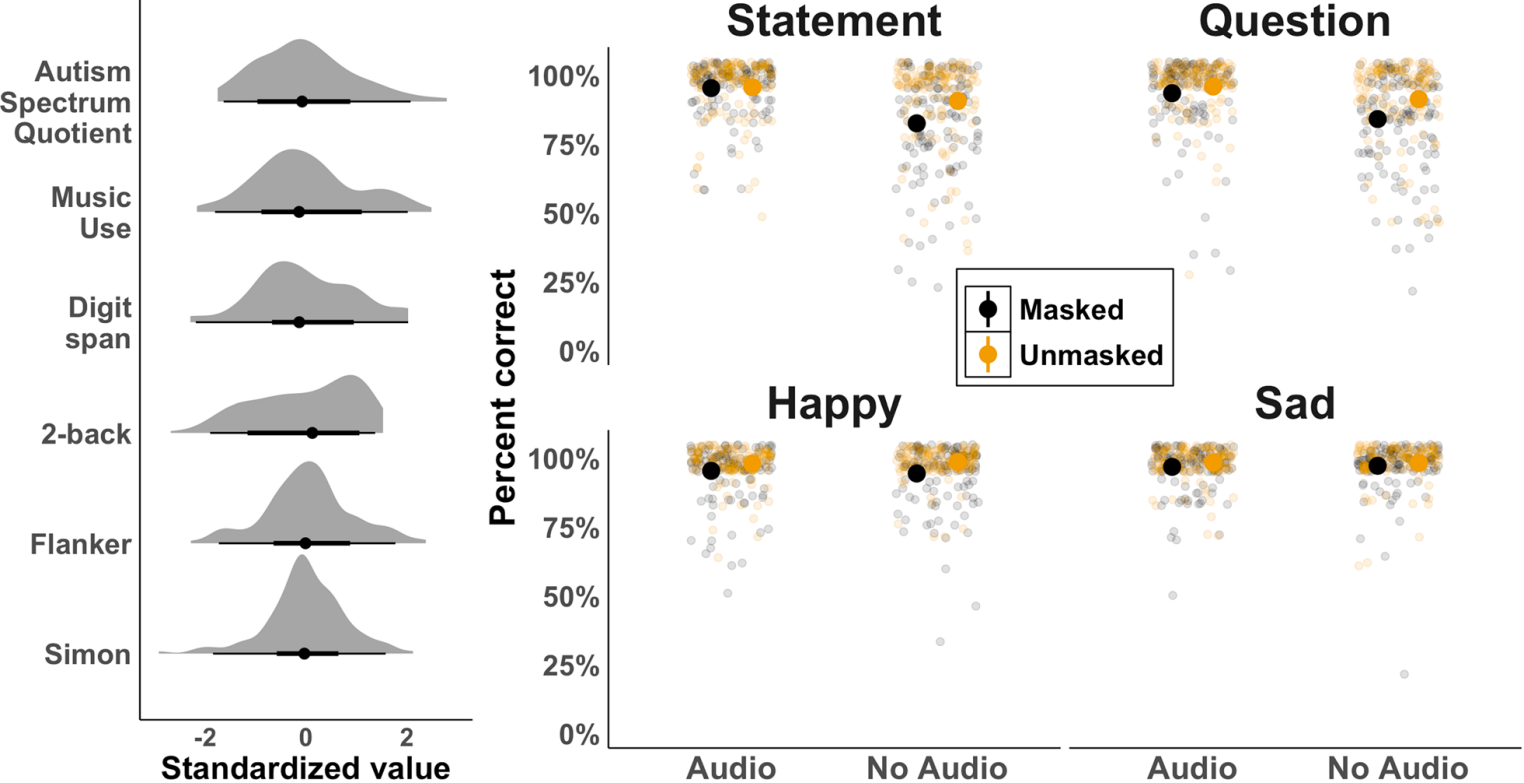
Standardized individual predictors (left) and performance on eight prosody–mask–audio conditions in the prosody task (right)

Figure 3 (left) plots the standardized results from the six individual differences tasks, all of which showed slightly different distributions. After correcting for multiple comparisons, none of the performances on the six variables were correlated with one another (*p*s > 0.05). Figure 3 (right) plots accuracy in the prosody task. Each point represents one participant’s mean given the eight prosody–mask–audio conditions. Condition means are plotted in the large points. For the majority of participants, the task was relatively easy, but for some, this was a difficult task.

**Fig. 4:**
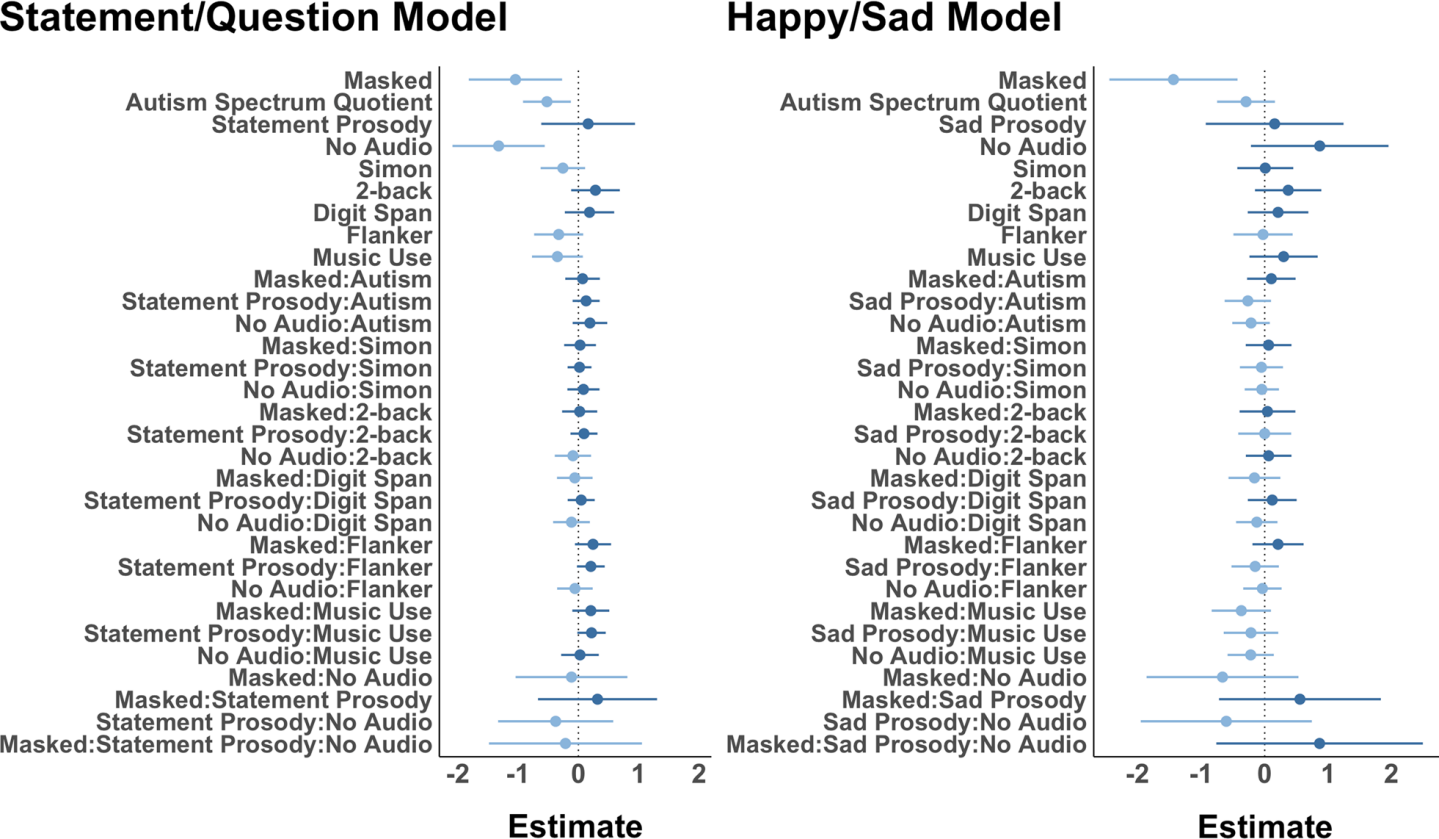
Mixed-effects logistic regression estimates for statement/question prosody model (left) and happy/sad prosody model (right). The plot shows the log-odds estimate along with 95% confidence intervals. Darker shade indicates a positive estimate; lighter shade indicates a negative estimate

Figure 4 plots the two models’ log-odds and 95% confidence intervals. For each estimate, the darker shade indicates a positive estimate whereas the lighter shade indicates a negative estimate. The statement/question model (conditional *R*^2^ = 0.53; BIC = 4441.2) revealed masked speech was harder to identify than unmasked speech (*ß* = − 1.04, SE = 0.39, *Z* = − 2.65, *p* = 0.008), and videos with no audio were harder to identify than videos with audio (*ß* = − 1.32, SE = 0.39, *Z* = − 3.39, *p* < 0.001). One individual difference predictor was found to be significant: Autism Spectrum Quotient (*ß* = − 0.52, SE = 0.20, *Z* = − 2.58, *p* = 0.01). All other predictors and interactions were null (*p*s > 0.05).

The happy/sad model (conditional *R*^2^ = 0.53; BIC = 2156.5) revealed masked speech was harder to identify than unmasked speech (*ß* = − 1.44, SE = 0.51, *Z* = − 2.79, *p* = 0.005). All other predictors and interactions were null (*p*s > 0.05).

In sum, for both models, masked speech significantly reduced the log-odds of correct identification. Autism Spectrum Quotient also reduced the log-odds of correct identification; however, this reduction was only statistically significant in the statement/question model. Neither model revealed a significant effect of prosody. The lack of audio resulted in a reduced log-odds of correct identification only in the statement/question model. All other predictors (and interactions) neither significantly increased nor decreased the log-odds of correct identification at an alpha level of 0.05.

## Discussion

This exploratory study set out to examine how face masks affect listeners’ perception of statement/question intonational prosody and happy/sad emotional prosody. We were specifically interested in six individual differences across participants and tested whether they predicted performance on the prosody task. We present three findings from our study.

First, we found that masks affected the discrimination of both statement/question and happy/sad prosody. Items produced with a mask were harder for participants to correctly discriminate than those produced without a mask. The acoustic and facial cues typically relied on for prosody discrimination were reduced as a result of the face mask, which in turn reduced listeners’ accuracy. This extends recent findings, which showed that masks do not necessarily affect individual word recognition accuracy in speech presented without background noise (Magee et al., 2020; Smiljanic et al., 2021; though see Brown et al., 2021; Toscano & Toscano, 2021 for speech presented in noise). Our results, however, indicate that masks *can* affect prosody discrimination of speech presented without background noise. These results also underscore how lexical access alone is not sufficient for understanding emotional and intonational prosody (Cutler, 2012). Importantly, as Fig. 3 (right) shows, we found a considerable range of behavior in our prosody discrimination task, which supports the observation that daily communication with masks is more challenging for some listeners than it is for others. We found this to be particularly true for perception of prosody when the speaker was masked.

Second, we found that the lack of audio only affected discrimination of statement/question prosody. These results are most likely because our speaker conveyed more facial cues while producing happy/sad prosody than statement/question prosody. Because the stimuli were recorded outside of a lab, we did not control facial cues as tightly as we would have preferred. In a post hoc exploratory analysis, we had 10 new participants rate the 128 videos for their facial movement (1—no movement; 5—full body/face movement). As expected, the statement/question videos were rated as having, on average, less facial movement than the happy/sad videos. These preliminary results suggest that facial cues were more helpful in determining the happy/sad contrast than they were in determining the statement/question contrast. Moreover, with enough facial cues, audio may not be a necessary condition for correct emotional prosody identification (e.g., Lansing & McConkie, 1999; Munhall et al., 2004). Indeed, while some participants struggled in our task, many of our participants did not make a mistake in the prosody task. For now, we are unable to say whether this difference in facial cues or something inherent to statement/question prosody caused the results and therefore refrain from further speculation.

Third, we observed high participant variability in our results in line with previous studies on the individual differences in prosody perception (e.g., Baumann & Winter, 2018; Roy et al., 2017). We found a general trend in that participants with higher Autism Spectrum Quotient scores (i.e., more autistic-like behavior) struggled to identify prosody correctly. This was a statistically significant effect for statement/question items but a nonsignificant effect for happy/sad items. These Autism Spectrum Quotient results, in part, support previous research on autism and prosody (e.g., McCann & Peppé, 2003; Philip et al., 2010; Paul et al., 2005), which has shown that individuals with autism spectrum disorder typically struggle to process emotional information quickly, including verbal and nonverbal emotional cues (e.g., Clark et al., 2008; Eack et al., 2015; Peppé et al., 2011). Autistic listeners also often struggle to perceive subtle acoustic differences such as *F*0 rise and fall, i.e., a primary cue in statements and questions (Järvinen-Pasley et al., 2008; though see Wang et al., 2021 for conflicting results). We also note that we did not find an interaction between Autism Spectrum Quotient and masks, which suggests that masks did not disproportionally affect those listeners with more autistic traits.

With regards to the other individual difference predictors, we found that music experience and use had no effect on performance in the prosody task. This is a somewhat unexpected finding as previous research has indicated that musical training and musical experience tends to result in an overall improved prosody perception, particularly in emotional and intonational prosody (e.g., Hausen et al., 2013; Lima & Castro, 2011; Thompson et al., 2004). This null effect may have been driven, in part, by the relatively high accuracy participants reached in the task. Therefore, any musical advantage may not have been needed given how easy the task was for participants.

We found that congruence task behavior (flanker, Simon) did not predict prosody task behavior. These results go against our initial prediction that performance on congruency tasks may predict masked prosody discrimination given that masks can be distracting to the listener (e.g., Carbon, 2020). This null effect may have been due to the relatively high accuracy participants reached in the prosody task and/or due to flanker and Simon tasks being more useful measures for studies on bilingualism and language switching rather than prosody perception (see Paap et al., 2017 for discussion).

We found no effects of phonological short-term memory (2-back, digit span) in either of our models. Because behavior on none of the tasks was correlated, it is possible that the tasks were measuring potentially different facets of the construct typically referred to as “working memory capacity” (see Conway et al., 2005). One likely explanation for the observed pattern of phonological short-term memory results is that neither task sufficiently involved linguistic awareness. The digit span task involved numeric awareness and the 2-back task did not sufficiently involve linguistic awareness given that participants only had to attend to a letter rather than a string of letters or word (Jacquemot & Scott, 2006; Jaeggi et al., 2010). Research has also called into question the use of the digit span task as a measure of attention or memory (Groth-Marnat & Baker, 2003).

Limitations to this study include the following: First, we did not record each prosody sentence with and without a mask. Presenting each sentence in both masked and unmasked conditions (across participants) would have been a preferable design choice, given that the sentences assigned to each mask condition may have differed in the strength of their prosodic cues, the extent to which they can be lip-read, etc. Second, the recorded happy/sad prosody was a simulated or portrayed prosody. In other words, our speaker was not actually happy or sad when producing the sentences. These emotional portrayals were based on stereotypical vocal expressions rather than psychophysiological effects on the voice (see Scherer, 2003). We note, however, that portrayed prosodies are typically recognized by listeners as the intended emotion and all emotions are, to some extent, “portrayals” given the sociocultural norms of speech and emotion (see Banse & Scherer, 1996 for additional discussion). Third, the recordings were made in a fixed order, which introduced a potential confound between recording order and prosody condition. For example, statements were always recorded before questions. It is unclear to what degree (if any) this recording procedure affected the results. We acknowledge that a better design would have been to counterbalance the order of the prosody condition such that half the statements were recorded first and half were recorded second. Fourth, because we did not alter the audio it is unclear whether acoustic differences, on their own, were enough to drive the differences in performance between the mask conditions. For example, it remains an open question whether accuracy would remain the same had the stimuli produced without a mask been altered to lower the *F*0 and amplitude to match the acoustic characteristics of the speech produced with a mask. We note that this would not change the fact that the masks still affect accuracy, but it would help us better pinpoint the reason for this accuracy decrease.

To conclude, our results indicate that higher-level understanding of intonations and emotions can be hindered by face masks. Speakers should keep in mind the listener and consider increasing pitch and volume in certain communicative contexts, if possible, when speaking with a mask. This may improve understanding. Increased pitch and volume may also be helpful when facial cues are obscured by the mask. Finally, speakers will come in contact with a wide variety of listeners. It is important to remember that not all listeners can detect subtle facial cues associated with emotions—even without masks. Speech communication may benefit from explicit statements of emotion. At the very least, by explicitly stating an emotion like “I am happy,” the listener will not wonder whether the speaker is smiling or frowning behind the mask.

## Data Availability

All data, materials, and code for analyses are available via the OSF at https://osf.io/gs79t/.
